# A Review of Formulation Strategies for Cyclodextrin-Enhanced Solid Lipid Nanoparticles (SLNs) and Nanostructured Lipid Carriers (NLCs)

**DOI:** 10.3390/ijms26136509

**Published:** 2025-07-06

**Authors:** Tarek Alloush, Burcu Demiralp

**Affiliations:** 1 Institute of Health Sciences, Istanbul University, Istanbul 34126, Türkiye; 2 Department of Pharmaceutical Technology, Faculty of Pharmacy, Istanbul University, Istanbul 34126, Türkiye; bmesut@istanbul.edu.tr

**Keywords:** cyclodextrins, solid lipid nanoparticles, nanostructured lipid carriers, poorly soluble drugs, inclusion complex

## Abstract

The advancement of efficient drug delivery systems continues to pose a significant problem in pharmaceutical sciences, especially for compounds with limited water solubility. Lipid-based systems, including solid lipid nanoparticles (SLNs) and nanostructured lipid carriers (NLCs), have emerged as viable options owing to their biocompatibility, capability to safeguard labile chemicals, and potential for prolonged release. Nonetheless, the encapsulation efficiency (EE) and release dynamics of these carriers can be enhanced by including cyclodextrins (CDs)—cyclic oligosaccharides recognized for their ability to form inclusion complexes with hydrophobic compounds. This article offers an extensive analysis of CD-modified SLNs and NLCs as multifunctional drug delivery systems. The article analyses the fundamental principles of these systems, highlighting the pre-complexation of the drug with cyclodextrins before lipid incorporation, co-encapsulation techniques, and surface adsorption after formulation. Attention is concentrated on the physicochemical interactions between cyclodextrins and lipid matrices, which influence essential factors such as particle size, encapsulation efficiency, and colloidal stability. The review includes characterization techniques, such as particle size analysis, zeta potential measurement, drug release studies, and Fourier-transform infrared spectroscopy (FT-IR)/Nuclear Magnetic Resonance (NMR) analyses. The study highlights the application of these systems across many routes of administration, including oral, topical, and mucosal, illustrating their adaptability and potential for targeted delivery. The review outlines current formulation challenges, including stability issues, drug leakage, and scalability concerns, and proposes solutions through advanced approaches, such as stimuli-responsive release mechanisms and computer modeling for system optimization. The study emphasizes the importance of regulatory aspects and outlines future directions in the development of CD-lipid hybrid nanocarriers, showcasing its potential to revolutionize the delivery of poorly soluble drugs.

## 1. Introduction

In pharmaceutical development, a significant number of emerging drug candidates continue to exhibit poor water solubility, particularly when intended for oral administration [1]. This inherent limitation hinders their absorption and reduces both bioavailability and therapeutic effectiveness [2]. To overcome these challenges, lipid-based delivery systems, such as SLNs and nanostructured lipid carriers (NLCs), have been introduced. These systems enhance drug solubility, stabilize sensitive compounds, and offer sustained or controlled release profiles [3,4]. SLNs, when stabilized with surfactants, are known for their scalability, safety, and ability to improve bioavailability, though they may present limitations in drug loading capacity. In contrast, NLCs are created by blending solid and liquid lipids, forming a more flexible lipid matrix that accommodates greater drug loading and minimizes leakage during storage [5,6,7].

Despite their advantages, NLCs also present certain limitations. High surfactant levels required for stabilization can cause irritation, and improper lipid composition may lead to particle growth or phase separation over time [8]. Moreover, some drug molecules may migrate to the particle surface, leading to an initial burst release or leakage during storage [8]. Integrating cyclodextrins into NLCs offers a promising strategy to overcome these drawbacks. CDs form inclusion complexes with drug molecules, keeping them molecularly dispersed and retained within the lipid matrix, which minimizes drug expulsion and burst release. Additionally, CDs enhance the aqueous stability of the dispersion by preventing drug crystallization and acting as a stabilizing interface. Together, these effects improve the overall stability, entrapment efficiency, and controlled-release performance of NLCs [9,10].

Cyclodextrins (CDs), classified as cyclic oligosaccharides, can encapsulate hydrophobic molecules within their cavity to form inclusion complexes. When combined with lipid carriers, CDs can influence encapsulation efficiency, colloidal stability, and the kinetics of drug release [11,12]. CDs are also applicable for topical formulations, especially in environments with variable pH, such as mucosal membranes and tumor tissues [13]. When integrated with lipid-based carriers, CDs contribute to enhanced drug entrapment, improved stability in aqueous media, and regulated release behavior. These hybrid systems effectively merge the solubilizing capacity of CDs with the functional benefits of SLNs and NLCs, making them promising platforms for formulating poorly soluble therapeutic agents [14,15].

Although multiple studies have explored the synergy between CDs and lipid-based nanoparticles, comprehensive analyses of formulation methodologies, underlying mechanisms, and comparative performance remain limited. This review aims to fill that gap by providing a detailed overview of the principles behind CD-SLN and CD-NLC systems. It emphasizes novel formulation strategies, structural characterization, and potential therapeutic uses. The review also discusses the molecular interactions between CDs, lipids, and drugs, highlighting their effects on particle characteristics, physical stability, and drug release dynamics—ultimately demonstrating how these systems can enhance drug delivery outcomes across various applications.

## 2. Fundamentals of Solid Lipid Nanoparticles (SLNs) and Nanostructured Lipid Carriers (NLCs)

SLNs and NLCs are colloidal carriers, measuring under one micron, employed for the administration of hydrophobic medicines through several ways [16]. Both methodologies employ biocompatible lipids, providing benefits like controlled drug release, stabilization of volatile substances, removal of organic solvents, and feasibility for large-scale manufacturing [17,18].

### 2.1. Solid Lipid Nanoparticles (SLNs)

Solid lipid nanoparticles first emerged in the early 1990s as a novel type of colloidal carrier, offering an alternative to systems like emulsions, liposomes, and polymer-based nanoparticles [19]. They are formulated using solid lipids—such as stearic acid, cetyl palmitate, or glyceryl behenate—that remain solid at both room and body temperature [20]. To ensure dispersion stability in aqueous environments, these lipids are combined with surface-active agents like poloxamers or polysorbates, which help stabilize the particle interface [21].

SLNs have a strong structure that can hold hydrophobic drugs inside their crystalline core [22]. Some of the good things about SLNs are the following [23,24]:

- Biocompatibility and biodegradability;
- Protection of encapsulated drugs from degradation;
- Avoidance of burst release associated with some other nanocarriers;
- Ease of surface modification for targeted delivery.

However, SLNs also have certain limitations. Because the lipid matrix has a lot of crystals, it is often hard to add drugs to it [25]. Also, during storage, recrystallization can happen, which might cause the medicine to be released and make it less stable over time [26].

### 2.2. Nanostructured Lipid Carriers (NLCs)

Nanostructured lipid carriers (NLCs) were developed as the second generation of lipid nanoparticles to overcome the limitations of SLNs [27]. These systems incorporate a mixture of solid and liquid lipids, with reported ratios ranging broadly from 60:40 to 90:10, depending on the formulation goals [8,28]. The inclusion of liquid lipids—such as medium-chain triglycerides or oleic acid—creates a less-ordered internal structure, introducing imperfections into the lipid matrix. These imperfections increase the space available for drug accommodation, thereby enhancing drug loading capacity and reducing the risk of drug expulsion [29,30]. Compared to SLNs, NLCs offer the following [31]:

- Higher drug loading capacity;
- Improved long-term physical stability;
- Reduced crystallinity and lower risk of polymorphic transitions;
- Greater flexibility in drug release kinetics.

**Figure 1:**
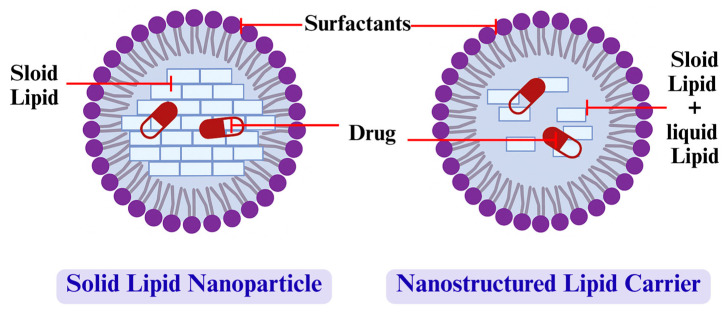
Schematic illustration of solid lipid nanoparticles (SLNs) and nanostructured lipid carriers (NLCs).

A schematic comparison of the internal structures of SLNs and NLCs is shown in Figure 1.

### 2.3. Preparation Methods

#### 2.3.1. Hot High-Pressure Homogenization (Hot HPH)

In this widely used method, the lipid and drug are melted (typically 5–10 °C above the lipid melting point) and emulsified into a hot aqueous surfactant solution using a high-pressure homogenizer at 500–1500 bar. The hot emulsion is then cooled to solidify the lipid into nanoparticles. Advantages include scalability and suitability for lipophilic drugs with high loading efficiency. Limitations include the possible degradation of heat-sensitive drugs and drug partitioning into the aqueous phase if homogenization is too vigorous [32,33].

#### 2.3.2. Cold High-Pressure Homogenization (Cold HPH)

Here, the drug–lipid melt is rapidly cooled (e.g., with liquid nitrogen) and milled into microparticles, which are then dispersed in a cold surfactant solution and homogenized at a low temperature (0–4 °C). This prevents drug migration and avoids thermal degradation, making it suitable for thermolabile and hydrophilic drugs. Limitations include broader particle size distributions and slightly lower process efficiency compared to hot HPH [32].

#### 2.3.3. Microemulsion Technique

A hot, clear oil-in-water microemulsion is formed by mixing molten lipid (with dissolved drug) into an aqueous surfactant/co-surfactant solution at elevated temperature. Rapid dilution with cold water (2–10 °C) then causes the lipid to crystallize into nanoparticles. This method is solvent-free and reproducible but requires high surfactant concentrations and large water volumes, necessitating post-process concentration [32,34].

#### 2.3.4. Emulsification–Solvent Evaporation

In this approach, the lipid and drug are dissolved in a water-immiscible organic solvent (e.g., dichloromethane) and emulsified into an aqueous surfactant phase. Solvent evaporation precipitates solid lipid nanoparticles. Advantages include suitability for heat-sensitive drugs. Limitations involve the complete removal of organic solvents and lower efficiency for hydrophilic drug loading [32,35].

#### 2.3.5. Emulsification–Solvent Diffusion

This variation is used for dissolving solid lipids (SLNs) or solid–liquid lipid mixtures (NLCs) in organic solvents, then emulsifying into an aqueous surfactant solution. Upon dilution or stirring, the solvent diffuses out or evaporates, causing lipid nanoparticle formation. It uses mild conditions and low energy but requires careful solvent removal to avoid toxicity [33,36].

#### 2.3.6. Solvent Injection (Nanoprecipitation)

Lipid and drug are dissolved in a water-miscible solvent (e.g., ethanol) and rapidly injected into an aqueous surfactant solution. The solvent diffuses away, causing the lipid to form nanoparticles. Advantages include simplicity, no heating, and suitability for heat-sensitive actives. Limitations include solvent removal steps and typically low final lipid concentrations [37].

#### 2.3.7. Coacervation (Fatty Acid Coacervation)

Fatty acid coacervation is a solvent-free, low-energy method for producing SLNs and NLCs, relying on the acidification of alkaline fatty acid soaps (e.g., sodium stearate) to trigger protonation and precipitation of solid nanoparticles. This environmentally friendly approach uses mild conditions and stabilizers like Polyvinyl Alcohol (PVA), supporting drug loading either during lipid dissolution or after nanoparticle formation. While limited to simple fatty-acid lipids capable of forming alkaline salts and unsuitable for pH-sensitive drugs, it has demonstrated practical utility by enabling insulin delivery with measurable bioavailability in rats and improving the anticancer activity of temozolomide-loaded SLNs without notable toxicity [38].

#### 2.3.8. Membrane Contactor Method

The membrane contactor method produces SLNs and NLCs by forcing a hot lipid phase through a porous membrane into an aqueous surfactant solution, forming fine droplets that solidify upon cooling. Particle size can be controlled via membrane pore size and process parameters. This scalable technique enables the production of formulations like vitamin E-loaded SLNs but requires specialized equipment and faces membrane clogging issues [38].

#### 2.3.9. Ultrasonication (High-Shear Homogenization)

High-frequency ultrasound breaks coarse emulsions into nanoscale droplets via cavitation. It is often combined with mechanical stirring. Advantages include simplicity, rapid processing, and solvent-free operation. Limitations include low solid yields, need for high surfactant levels, and broader size distributions [32].

#### 2.3.10. Spray Drying

A dispersion of SLNs or NLCs is atomized into a hot drying chamber to produce dry powder. Advantages include ease of scale-up, solid-state stability, and potential for inhalable formulations. Limitations involve heat-induced drug degradation or aggregation, requiring formulation additives for stabilization [33,39].

#### 2.3.11. Supercritical Fluid Techniques

Supercritical CO_2_ acts as a solvent or antisolvent to form lipid nanoparticles via rapid expansion or spraying. Advantages include solvent-free, residue-free processing with tunable properties. Limitations involve expensive equipment, complex control, and challenges in producing uniform nanoscale particles [33,38].

#### 2.3.12. Phase Inversion Temperature (PIT) Method

The phase inversion temperature (PIT) method enables the solvent-free production of SLNs and NLCs by exploiting the temperature-dependent behavior of non-ionic surfactants, whose hydrophilic-lipophilic balance (HLB) changes with temperature [40,41]. At elevated temperatures exceeding the PIT, W/O emulsions form due to reduced surfactant hydration and affinity shifts between aqueous and lipid phases [42,43]. Rapid cooling then inverts the emulsion to O/W, causing lipid droplets to solidify into nanoparticles [44,45]. This approach yields small particle sizes, narrow distributions, and high drug loading potential, and has been successfully applied to prepare various drug-loaded NLCs with improved stability and release profiles [46,47]. However, it demands precise temperature control, may involve multiple heating-cooling cycles (e.g., 60–90 °C), and can result in low nanoemulsion stability [48,49].

#### 2.3.13. Double Emulsion (W/O/W)

The double emulsion (W/O/W) method is widely used to encapsulate hydrophilic drugs and biomolecules into SLNs and NLCs by forming a primary water-in-oil emulsion that is re-emulsified in water to create a W/O/W system [50,51]. During solvent removal or cooling, lipid droplets solidify into nanoparticles, enabling the delivery of water-soluble actives [52,53]. Advantages include protection of hydrophilic compounds and moderate entrapment, while limitations involve multiple processing steps, risk of emulsion breakdown, and often relatively low drug loading and larger particle sizes [54,55].

#### 2.3.14. Electrospray (Electrohydrodynamic Atomization)

A lipid solution is fed through a capillary under high voltage, creating a fine spray of charged droplets that dry into nanoparticles. Advantages include uniform particle production and the avoidance of heat. Limitations include low throughput, high-voltage requirements, and scale-up challenges [56,57].

#### 2.3.15. Microfluidic Mixing

Lipid and aqueous phases are pumped through microscale channels at controlled flow rates, mixing rapidly under laminar conditions to form nanoparticles. Advantages include precise size control and narrow distributions. Limitations involve specialized equipment, risk of clogging, and limited single-chip throughput (though parallelization is possible) [5].

#### 2.3.16. Microfluidization

An advanced high-energy technique related to HPH, microfluidization uses specific interaction chambers to force the emulsion through microchannels at high pressures. This produces extremely fine, uniform nanoparticles with narrow size distributions. Advantages include excellent reproducibility and scalability. Limitations involve high equipment costs and maintenance requirements [58,59].

#### 2.3.17. Spontaneous Emulsification

This is a low-energy method where the lipid phase with surfactant is mixed at elevated temperature and then introduced into aqueous phase under mild stirring without external energy input. Nanoparticles form spontaneously due to interfacial turbulence and solvent diffusion. Advantages include mild conditions and no high-pressure equipment. Limitations include dependence on solvent selection and typically lower encapsulation efficiencies [60,61].

#### 2.3.18. High-Speed Homogenization

High-speed homogenization uses rapid mechanical stirring to apply shear forces that disperse the lipid phase into the aqueous surfactant solution. It is simple and cost-effective but typically produces larger, more polydisperse particles than high-pressure or ultrasonic methods. Often, it serves as a pre-emulsification step before ultra-sonication to achieve finer, more uniform nanoparticles [5].

#### 2.3.19. Microwave-Assisted Synthesis (MAS)

This green, energy-efficient method uses microwave radiation to rapidly and uniformly heat the reaction medium, promoting lipid melting and nanoparticle formation. In MAS, microwaves couple directly with particles in the formulation container, enabling rapid dielectric heating independent of vessel conductivity and avoiding dipole polarization or ionic conductance [62]. Advantages include fast reaction times, improved particle size control, greater homogeneity, and potential reduction in solvent use. Limitations involve equipment costs and careful optimization to prevent hot spots or degradation [63].

#### 2.3.20. Ultrasound-Assisted Synthesis (UAS)

This method uses acoustic cavitation to promote lipid melting and nanoparticle formation, improving nucleation, reducing reaction times, and controlling particle size. Advantages include efficient encapsulation, green processing, and suitability for emulsion manufacturing. Limitations involve equipment optimization and scale-up challenges [63,64].

## 3. Cyclodextrins: Properties and Pharmaceutical Relevance

**Figure 2:**
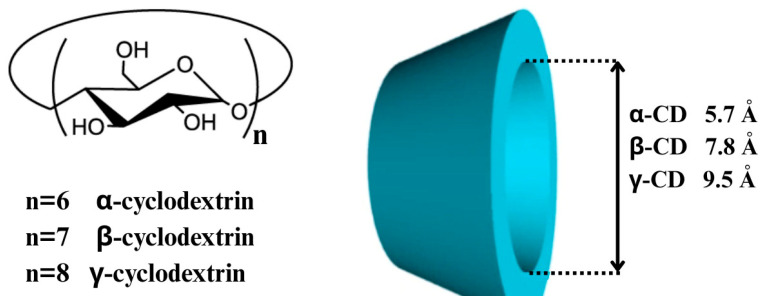
Illustrates the differences between the three main types of cyclodextrins.

Cyclodextrins (CDs) are a group of naturally occurring, enzymatically derived cyclic oligosaccharides composed of α-(1→4)-linked D-glucopyranose units [65]. They are primarily produced through the enzymatic degradation of starch by cyclodextrin glycosyltransferase (CGTase) [66]. The three most common native CDs (Figure 2) are α-cyclodextrin (six glucose units), β-cyclodextrin (seven glucose units), and γ-cyclodextrin (eight glucose units) [67]. These molecules possess a truncated cone-shaped architecture, characterized by a hydrophilic exterior—attributable to the presence of multiple hydroxyl groups—and a relatively hydrophobic inner cavity [68]. The unique molecular architecture of CDs enables them to form non-covalent inclusion complexes with a wide range of poorly water-soluble compounds [69]. The guest molecule is accommodated in the apolar cavity of the CD, leading to modifications in its physicochemical and pharmacokinetic properties [70]. The driving forces for this host–guest interaction include van der Waals forces, hydrogen bonding, hydrophobic interactions, and the displacement of high-energy water molecules from the cavity [71].

### 3.1. Pharmaceutical Utility

Cyclodextrins are widely employed in pharmaceutical formulations to enhance the aqueous solubility, chemical stability, and bioavailability of active pharmaceutical ingredients (APIs), particularly those classified as Biopharmaceutical Classification System (BCS) Class II and IV drugs [72,73]. Their inclusion complexes not only improve drug dissolution rates but also help in stabilizing labile drugs against hydrolysis, oxidation, photodegradation, and thermal decomposition [74]. CDs can also mask unpleasant tastes and odors, reduce gastrointestinal or injection site irritation, and enhance the permeability of drugs across mucosal membranes [75,76,77,78].

The versatility of CDs extends across various routes of administration, including oral, parenteral, ocular, nasal, pulmonary, buccal, and transdermal delivery systems [79]. In the oral route, for instance, CDs facilitate rapid dissolution in the gastrointestinal tract, thereby improving absorption [80]. In parenteral applications, modified CDs, such as sulfobutylether-β-cyclodextrin (SBE-β-CD) and hydroxypropyl-β-cyclodextrin (HP-β-CD), are employed due to their higher aqueous solubility and lower nephrotoxicity [81]. In topical systems, CDs can enhance drug penetration through the stratum corneum by disrupting lipid packing or forming supersaturated drug solutions [9,82].

### 3.2. Modified Cyclodextrins and Regulatory Status

To overcome the limitations of native CDs—such as limited solubility (particularly for β-CD) and potential nephrotoxicity—numerous chemically modified derivatives have been developed [83]. These include hydroxyalkylated CDs (e.g., HP-β-CD, Hydroxypropyl-γ-Cyclodextrin (HP-γ-CD)), methylated CDs (e.g., RM-β-CD, DM-β-CD), and charged derivatives (e.g., SBE-β-CD). These modifications improve complexation efficiency, reduce crystallinity and enhance safety profiles [84,85,86].

Many CD-based products have received regulatory approval and are commercially available in various therapeutic categories, such as antifungals (e.g., itraconazole), anti-inflammatories (e.g., diclofenac), and anticancer agents (e.g., docetaxel) [87,88,89]. Regulatory agencies, including the Food and Drug Administration (FDA) and European Medicines Agency (EMA) have provided monographs for selected CDs in pharmacopeias, recognizing them as generally safe when used within specified limits [90]. Native cyclodextrins (CDs) can be modified into more than 1500 derivatives, although only a limited number have received FDA approval for human application. These comprise 2-hydroxypropyl-β-cyclodextrin (HPβCD), sanctioned for oral and intravenous administration, and 2-hydroxypropyl-γ-cyclodextrin (HPγCD), authorized for topical use at a dose of 1.5%. Sulfobutyl ether β-cyclodextrin (SBEβCD) is FDA-approved for both oral and intravenous administration, recognized for its non-nephrotoxic characteristics [91].

Table 1 summarizes selected modified cyclodextrins approved for human pharmaceutical use, highlighting their chemical modifications, solubility, routes of administration, and representative applications.

### 3.3. Integration with Nanocarrier Systems

Recent advances in nanotechnology have expanded the application of CDs from standalone excipients to components of multifunctional drug delivery systems [101]. When integrated into lipid-based nanocarriers, such as solid lipid nanoparticles (SLNs) and nanostructured lipid carriers (NLCs), CDs can further enhance formulation performance [102]. They may serve in multiple roles: as solubilizing agents through pre-complexation, as matrix stabilizers when included within the lipid phase, or as surface modifiers to improve mucoadhesiveness and site-specific targeting [103,104,105].

The amalgamation of cyclodextrins with lipid nanoparticles produces hybrid delivery systems that utilize the solubilization efficacy of cyclodextrins together with the biocompatibility, protective attributes, and controlled-release functionalities of lipid carriers [106,107,108,109]. These systems have exhibited synergistic advantages in strengthening encapsulation efficiency, extending drug release, minimizing burst effects, and improving pharmacokinetics [110,111].

## 4. Formulation Strategies for Cyclodextrin-Modified SLNs and NLCs

The effective formulation of cyclodextrin (CD)-modified solid lipid nanoparticles (SLNs) and nanostructured lipid carriers (NLCs) depends on optimized techniques that consider drug–CD interactions, lipid compatibility, processing conditions, and the desired route of administration. This section discusses the principal formulation processes and experimental factors pertinent to the creation of CD-lipid hybrid systems, focusing on optimizing drug entrapment, physical stability, and therapeutic efficacy.

### 4.1. Pre-Formulation Studies

Before designing a CD–SLN or CD–NLC system, pre-formulation work is critical to assess the following:

- Phase solubility behavior of the drug with various CDs to determine complexation efficiency and stability constants [112];
- Thermal stability of the CD–drug complex using Differential Scanning Calorimetry (DSC) and Thermogravimetric Analysis (TGA);
- Compatibility with lipids through miscibility studies;
- Complex characterization via FT-IR, X-ray Diffraction (XRD), and NMR to confirm inclusion [113].

These studies provide essential guidance on whether to proceed with pre-complexation or direct inclusion of the drug and CD into the lipid system.

### 4.2. Preparation Methods for Cyclodextrin-Modified SLNs and NLCs

Cyclodextrin-enhanced lipid nanocarriers represent a synergistic approach that combines the solubilization and stabilization potential of cyclodextrins (CDs) with the biocompatibility and controlled release capabilities of lipid-based systems. To fully exploit the benefits of this hybrid platform, several formulation strategies have been developed, each tailored to specific therapeutic goals and physicochemical characteristics of the active pharmaceutical ingredient (API) [114].

#### 4.2.1. Pre-Complexation Method

One common strategy involves the pre-complexation of the drug with a cyclodextrin, typically in aqueous or hydroalcoholic media, prior to its incorporation into the lipid carrier during the preparation of SLNs or NLCs. Pires et al. demonstrated that incorporating the cyclodextrin–essential oil complex into the lipid phase of NLCs significantly slowed drug release, reducing the burst effect and yielding Higuchi kinetics indicative of diffusion-controlled release and improved stability [14]. In contrast, Cirri et al. reported that incorporating hydrochlorothiazide–HPβCD complexes into the aqueous phase of NLCs produced a markedly faster release, with about 60% of the drug released within 30 min, compared to 6 h for uncomplexed drug-loaded NLCs [115]. These findings illustrate that the incorporation route strongly affects the release kinetics and can be tailored to achieve either sustained or immediate release profiles, depending on formulation goals. Depending on the method, the resulting inclusion complex may be dispersed into the lipid melt or the aqueous surfactant phase. Pre-complexation can enhance the solubility of poorly water-soluble drugs and significantly improve entrapment efficiency.

#### 4.2.2. Co-Encapsulation Method

Alternatively, the co-encapsulation of free cyclodextrins can be employed to achieve in situ complexation or to modulate drug release kinetics. In this strategy, CDs are added directly to the aqueous phase—without prior drug complexation—allowing them to interact with the drug during or after particle formation. This approach has been shown to influence the internal organization and viscosity of the nanoparticle matrix, potentially facilitating sustained drug release and improving colloidal stability [116].

#### 4.2.3. Surface Functionalization Method

Cyclodextrins may be employed post-formulation for nanoparticle surface modification. For example, Permana et al. demonstrated that SLNs containing plant extract were first prepared using the emulsion–solvent evaporation technique, then mixed post-formulation with a 1% HPβCD solution at 4 °C and gently stirred for 4 h to achieve surface modification. Excess unbound materials were removed by centrifugation using a 12 kDa cut-off membrane, confirming effective post-synthesis coating [117].

Similarly, Parvez et al. described the preparation of dual-drug-loaded SLNs modified with 2-hydroxypropyl-β-cyclodextrin (HPCD) via post-formulation incubation and freeze-drying, achieving stable surface modification that improved oral bioavailability and reduced toxicity in a murine model [108].

Surface functionalization using cyclodextrin derivatives—particularly methylated, thiolated, or hydroxypropyl β-CDs—represents an advanced strategy to enhance mucoadhesive interactions, prolong systemic circulation, and support targeted delivery. These derivatives can be covalently attached or adsorbed onto nanoparticle surfaces, facilitating tailored interactions with biological barriers, including mucosal and transdermal tissues [118,119,120]. Baek et al. further demonstrated that surface modification of paclitaxel-loaded SLNs with HPβCD improved aqueous solubility, cellular uptake, and lymphatic transport after oral administration [121].

Amphiphilic cyclodextrin-based nanoparticles also demonstrate such functionalities through charge-based modifications and in vivo drug targeting [122]. The influence of processing factors—including homogenization speed, sonication duration, and temperature—is essential for the efficacy of CD–lipid hybrid formulations. High-shear homogenization and ultrasonication are extensively utilized to reduce particle size and achieve uniform dispersion [123,124].

Emulsification energy (EEn), lipid composition, and surfactant type play critical roles in determining nanoparticle characteristics. Increasing EEn, such as using higher homogenization speeds or additional cycles, generates stronger shear forces that reduce particle size distribution (PSD), producing smaller, more uniform nanoparticles with improved stability; however, excessive energy may risk destabilizing the emulsion or degrading sensitive actives, and may even reduce encapsulation efficiency (EE) if leakage occurs [125,126]. Lipid composition influences the internal structure and drug loading: incorporating liquid lipids reduces matrix crystallinity, facilitating higher EE by improving drug solubility and often producing smaller PSD, while solid lipids can improve zeta potential (ZP) stability but may limit EE due to lower solubility compatibility. Surfactant type critically affects interfacial properties; surfactants with a suitable hydrophilic–lipophilic balance (HLB) optimize EE by enhancing drug partitioning into the lipid phase and stabilizing the emulsion, while ionic surfactants can increase the magnitude of ZP, improving colloidal stability and preventing aggregation [125,126].

### 4.3. Drug Selection Criteria for CD-Modified SLNs and NLCs

Cyclodextrin-modified solid lipid nanoparticles (SLNs) and nanostructured lipid carriers (NLCs) are particularly well-suited for drugs with specific physicochemical properties that benefit from both inclusion complexation and lipid encapsulation. Careful selection of the active pharmaceutical ingredient (API) is essential to ensure successful formulation, efficient drug loading, and improved therapeutic outcomes.

One of the primary criteria is poor aqueous solubility, characteristic of BCS Class II or IV drugs. These compounds typically dissolve only sparingly in water (often <100 µg/mL), limiting their bioavailability in conventional dosage forms. Incorporating cyclodextrins improves aqueous solubility through inclusion complex formation, while the lipid matrix provides additional stabilization and controlled release. Example: Paclitaxel has water solubility around 0.3 µg/mL and is classified as BCS Class IV, making it an excellent candidate for CD-lipid hybrid systems [121].

Another critical factor is lipophilicity, typically indicated by a Log *p* value greater than −2. Drugs with sufficient lipophilicity can partition effectively into the lipid core of nanoparticles while also forming stable inclusion complexes with cyclodextrins. This dual affinity facilitates both improved aqueous solubility and sustained release. For example, paclitaxel (Log *p* ≈ 3.9) is a BCS Class IV drug with extremely low solubility, making it an ideal candidate for CD–lipid hybrid systems that combine cyclodextrin complexation to enhance solubility and lipid encapsulation to control release [127]. Similarly, ibuprofen (Log *p* ≈ 3.5) has demonstrated improved dissolution and bioavailability when formulated as a cyclodextrin inclusion complex, supporting its use in CD-modified delivery systems [128]. Curcumin (Log *p* ≈ 3) is another widely studied lipophilic compound effectively delivered using cyclodextrin–lipid nanocarriers.

Molecular weight is another important consideration. Low molecular weight (100–500 Da) and low dose (5–50 mg) drugs are good candidates for CD complexation to improve both solubility and dissolution. In aqueous solutions, CDs can form inclusion, non-inclusion complexes, and hydrogen bond formation with adjacent CD molecules [129]. However, the extent of solubility enhancement is limited by the drug molecular weight and CD concentration. For instance, a 1:1 complex between drug (Molecular Weight (MW) 350 Da) and CD (0.1 M) can dissolve only up to 0.1 M drug or 35 mg ml^−1^ (assuming the K value is infinitely large). Thus, the possible enhancement in solubility can be predicted before performing the experiment.

Another important criterion is potency and the required dose. Nanoparticle systems have limited payload capacity, making them most suitable for drugs effective at relatively low daily doses (typically milligram-scale). Amphotericin B is a classic example: despite its potent systemic antifungal activity, its poor solubility and dose-limiting toxicity are significant challenges. Incorporating Amphotericin B into SLNs and NLCs has been shown to improve its molecular dispersion and reduce toxicity while enabling safer parenteral administration [130].

In summary, the optimal candidates for CD-modified SLN and NLC formulations are poorly water-soluble, sufficiently lipophilic, small enough for cyclodextrin complexation, and potent at low doses. These systems effectively combine the solubilizing power of CDs with the protective and controlled-release advantages of lipid nanocarriers, offering an advanced platform for delivering otherwise challenging drug molecules.

## 5. Characterization Techniques for Cyclodextrin-Modified SLNs and NLCs

Thorough physicochemical evaluation is crucial for determining the success of modified solid lipid nanoparticles (SLNs) and nanostructured lipid carriers (NLCs). Incorporating cyclodextrins (CDs) can significantly affect the fundamental characteristics of these systems [102]. To ensure optimal functionality and reproducibility, it is important to deeply investigate formulation behavior, drug release mechanisms, particle size, and surface properties. This section outlines the key analytical techniques—both traditional and advanced—used to characterize CD-integrated SLN and NLC formulations.

### 5.1. Particle Size and Polydispersity Index (PDI)

Dynamic Light Scattering (DLS) is the primary method for ascertaining the hydrodynamic diameter and polydispersity index (PDI) of nanoparticles. The dimensions of particles significantly influence medication release, stability, and biological distribution.

- Expected range: <200 nm.
- PDI: Ideally < 0.3 for monodisperse systems [131].
- Cyclodextrin inclusion may result in a modest increase in size due to modified surface characteristics or aggregation generated by complexation [132].

### 5.2. Zeta Potential

Zeta potential, usually evaluated using electrophoretic light scattering, indicates the surface charge and colloidal stability of nanoparticles.

- High absolute values (±30 mV or more) indicate good stability [133].
- CD modification may shift the zeta potential depending on the CD type (neutral, cationic, or anionic) and its mode of incorporation (surface adsorption vs. internal complexation) [134].
- For example, anionic derivatives, such as SBE-β-CD, can increase the negative charge of the nanoparticles [135].

### 5.3. Entrapment Efficiency (EE%) and Drug Loading (DL%)

Entrapment efficiency and drug loading are crucial for evaluating the formulation’s capacity to carry and retain the active compound.

- EE% = [(Total drug − Free drug)/Total drug] × 100
- DL% = [(Entrapped drug/Total weight of nanoparticles)] × 100 [136,137]

Ultracentrifugation, dialysis, or filtration is typically used to separate free drug, followed by High-Performance Liquid Chromatography (HPLC) or Ultraviolet–Visible (UV–VIS) spectroscopy for quantification [138].

Pre-complexation with CDs often improves EE% by increasing drug solubility in the lipid phase or reducing drug crystallization during formulation [70,139].

### 5.4. Thermal Analysis

#### 5.4.1. Differential Scanning Calorimetry (DSC)

DSC evaluates thermal transitions, including melting points of lipids and possible interactions between drug, CD, and lipid matrix.

- Disappearance or shift of the drug’s melting peak may indicate successful encapsulation or complexation;
- Reduced enthalpy values suggest a decrease in lipid crystallinity, often enhanced by the presence of CDs [140].

#### 5.4.2. Thermogravimetric Analysis (TGA)

TGA assesses thermal stability and moisture content, particularly important for lyophilized systems [141].

### 5.5. Crystallinity and Structural Analysis

#### 5.5.1. X-Ray Diffraction (XRD)

XRD is used to assess the crystalline nature of the lipid matrix and the drug.

- A loss or reduction in drug-specific peaks indicates amorphization due to inclusion or encapsulation [142].
- CDs can reduce overall crystallinity, supporting better drug retention in the matrix [70].

#### 5.5.2. Fourier-Transform Infrared Spectroscopy (FT-IR)

FT-IR provides information about molecular interactions among drug, CD, and lipid [143].

- Shifts in characteristic peaks (e.g., carbonyl, hydroxyl) indicate hydrogen bonding or hydrophobic interactions.
- Used to confirm inclusion complex formation and detect chemical compatibility [11].

#### 5.5.3. Proton Nuclear Magnetic Resonance (^1^H-NMR)

^1^H-NMR helps elucidate the structural configuration of CD–drug complexes and verify the inclusion mode (internal vs. external). Shifts in proton resonance, particularly for CD cavity protons (H3 and H5), provide evidence of complexation [144].

### 5.6. Morphology and Surface Topography

Transmission electron microscopy (TEM) and scanning electron microscopy (SEM) provide high-resolution imaging without direct contact between the source and the sample. These techniques typically require the use of a high vacuum environment and involve specific sample preparation steps. While SEM is primarily used to visualize surface morphology, TEM offers detailed insight into the internal structure of the sample [145].

### 5.7. In Vitro Drug Release Studies

Drug release studies are typically performed using dialysis bag methods, United States Pharmacopeia (USP) dissolution apparatus, or Franz diffusion cells to simulate physiological conditions and evaluate the release-modulating effects of cyclodextrins (CDs) [146]. CD–SLN/NLC systems frequently exhibit a biphasic release profile, comprising an initial burst phase followed by sustained drug release [147]. The burst release is primarily due to drug molecules adsorbed on the nanoparticle surface or loosely associated with unstable CD complexes. In contrast, the sustained phase reflects diffusion-controlled release of drug molecules entrapped within the lipid matrix or stabilized by stronger CD–drug inclusion complexes. The physicochemical interactions between CDs and both the drug and lipid core significantly influence this release pattern. For example, Cavalli et al. reported that incorporation of β-cyclodextrin into hydrocortisone-loaded SLNs resulted in a slower and more prolonged release, attributed to stronger CD–drug binding and reduced drug diffusion from the core [107]. Furthermore, CDs can reduce lipid crystallinity, creating amorphous microdomains or diffusion barriers that further modulate release behavior. Such structural modifications also affect kinetic modeling; drug release from CD-modified systems is often best described by Higuchi or Korsmeyer–Peppas equations, indicating a combination of diffusion and erosion mechanisms [148].

The selection of a suitable kinetic model provides further insight into the dominant release mechanism. Zero-order kinetics, indicating a constant release rate, are rarely observed but may occur in matrix systems with sustained erosion. First-order models apply when drug release is concentration-dependent. The Higuchi model, which assumes Fickian diffusion, is widely used for lipid-based carriers including CD-modified systems [149]. The Korsmeyer–Peppas model is particularly relevant to CD–SLNs/NLCs, as it accommodates both diffusion and matrix relaxation mechanisms. The release exponent (n) helps distinguish between Fickian (n ≤ 0.43), non-Fickian (0.43 < n < 0.85), and case-II transport (n ≥ 0.85), providing a deeper understanding of the release behavior [150]. The inclusion of CDs often shifts the release profile toward diffusion-controlled mechanisms by stabilizing drug localization and modifying the matrix structure [14,103].

Additionally, formulation composition plays a critical role in controlling the initial burst. In SLNs, highly crystalline lipid cores can expel the drug to the surface, while NLCs with higher ratios of liquid lipids form more amorphous matrices that better retain the drug internally and reduce burst release. However, excessive liquid lipids can make the core too fluid, potentially increasing early diffusion. The amorphous form of the drug in CD complexes can also contribute to burst if surface-localized complexes dissolve quickly upon hydration [36]. For example, Baek et al. observed a modest initial burst from SLNs with HP-βCD–paclitaxel complexes at the surface, highlighting the need to balance matrix composition and CD complexation to minimize burst release and achieve sustained delivery [121].

### 5.8. Stability Studies

Stability testing assesses the physical and chemical integrity of formulations over time. Parameters include particle size, zeta potential, drug content, and appearance under various storage conditions (e.g., 4 °C, 25 °C, 40 °C) [151]. CD inclusion often enhances physical stability by inhibiting drug recrystallization and preventing nanoparticle aggregation [152].

An effective analytical toolset is crucial for characterizing CD-modified SLNs and NLCs. The integration of traditional methods (size, charge, drug loading) with sophisticated structural and interaction investigations (DSC, XRD, FT-IR, NMR) facilitates a thorough comprehension of these intricate systems.

## 6. Therapeutic Applications of Cyclodextrin-Enhanced Lipid Nanoparticles

Cyclodextrin–lipid hybrid nanocarriers, including solid lipid nanoparticles (SLNs) and nanostructured lipid carriers (NLCs), represent a promising platform for improving the delivery of poorly soluble or unstable pharmaceuticals. By combining cyclodextrin inclusion complexation with lipid-based delivery, these systems enhance solubility, increase drug loading and entrapment efficiency, and enable controlled or sustained release profiles—ultimately supporting better therapeutic outcomes with reduced systemic toxicity [121,147].

Cyclodextrin-modified SLNs and NLCs have been employed to increase the oral bioavailability of poorly soluble drugs by enhancing aqueous solubility and maintaining formulation stability under gastrointestinal conditions. For example, Baek et al. reported that incorporating HPβCD into paclitaxel-loaded SLNs improved the drug’s solubility from 0.3 μg/mL (pure drug) to 4.98 ± 0.18 μg/mL (−17-fold increase overall), and slightly over 11% higher than SLNs without cyclodextrin. The encapsulation efficiency (EE%) increased from 66.20 ± 0.14% to 71.02 ± 0.70%, indicating improved entrapment within the lipid matrix [121]. Similarly, Gidwani et al. showed that complexing altretamine with epichlorohydrin-β-cyclodextrin before SLN formulation increased solubility from 0.056 mg/mL to 1.083 mg/mL (−19-fold enhancement), raised drug loading from 11.29% to 14.36% (−27% increase), and improved EE% from 74.51% to 82.67%, confirming the role of CDs in boosting formulation performance.

For transdermal and dermal applications, CD–lipid nanocarriers improve the delivery of poorly soluble or volatile compounds, such as essential oils, by enhancing solubility and enabling targeted delivery to skin appendages. Pires et al. demonstrated that βCD-modified NLCs containing thyme oil achieved sustained release profiles and enhanced skin permeation, supporting effective antimicrobial delivery with potential for follicular targeting [14].

Cyclodextrins combined with lipid nanoparticles improve corneal drug deposition, retention, and formulation stability for ocular therapies. For instance, Liu et al. reported that HPβCD complexation in lutein-loaded NLCs increased solubility from 0.64 ± 0.02 μg/mL to 10.89 ± 0.16 μg/mL (−17-fold increase). Drug loading improved from 0.32 ± 0.01% to 0.47 ± 0.02% (−47% increase), while encapsulation efficiency rose from 91.8 ± 0.7% to 94.4 ± 0.5%, indicating the role of CDs in enhancing solubility, drug incorporation, and formulation stability [153].

Cyclodextrin-based SLNs have also been explored for oral anticancer drug delivery. By improving aqueous solubility, increasing drug loading and entrapment efficiency, and enabling prolonged release, these systems help achieve higher therapeutic efficacy with reduced systemic toxicity [147]. For example, the use of epichlorohydrin-β-cyclodextrin complexes in altretamine SLNs significantly improved solubility and formulation stability, demonstrating the benefit of CD–lipid hybrid approaches in oncology.

Table 2 summarizes representative therapeutic applications of cyclodextrin-modified SLNs and NLCs, organized by route of administration and formulation goals. These examples highlight how CD functionalization can improve solubility, bioavailability, targeting, and controlled release profiles across diverse pharmaceutical applications.

## 7. Challenges and Limitations of Cyclodextrin–Lipid Hybrid Systems

Despite their potential in enhancing solubility, stability, and bioavailability, cyclodextrin (CD)-modified solid lipid nanoparticles (SLNs) and nanostructured lipid carriers (NLCs) face several limitations that must be addressed for successful formulation and clinical application.

### 7.1. Formulation Complexity

The formulation of CD–lipid systems requires the careful consideration of multiple parameters, such as the drug-to-CD molar ratio, lipid composition, and the method of CD incorporation. Improper formulation may result in reduced drug permeation or limited complexation efficiency. Cyclodextrins only enhance the permeation of drugs that readily form inclusion complexes and can permeate the membrane once in contact. Hydrophilic CDs improve delivery primarily when the resistance of the unstirred water layer (UWL) is comparable to or greater than that of the membrane barrier. In contrast, lipophilic CDs may alter membrane characteristics. Therefore, achieving effective drug delivery with CD–lipid systems depends heavily on the drug’s ability to form complexes and the nature of the biological barrier [78].

### 7.2. Drug Loading Efficiency

While NLCs offer a higher drug loading capacity than SLNs, the inclusion of CDs can sometimes reduce entrapment efficiency due to a weak interaction between the drug–CD complex and the lipid matrix or partitioning of the drug into the aqueous phase [154,155].

### 7.3. Manufacturing and Scale-Up

Scaling up solid lipid nanoparticles (SLNs) involves challenges, such as maintaining particle uniformity, reproducibility between batches, and adapting production equipment, despite the use of Generally Recognized as Safe (GRAS)-status excipients and techniques offering extended shelf life and enhanced drug stability [111]. Additionally, the functional performance of cyclodextrins (CDs) can vary depending on their type and substitution pattern, which may affect their behavior as permeability enhancers and result in variability in drug delivery outcomes [3,78].

On a commercial scale, the manufacturing of NLCs—including those incorporating cyclodextrin–drug complexes—often requires larger solvent volumes for emulsification or co-solubilization steps, increasing cost and environmental burden [156,157]. Additionally, CD–drug complexes may increase the effective molecular weight of the encapsulated entity, potentially requiring higher lipid excipient content to maintain entrapment efficiency and stable dispersion. Such factors reduce the overall drug-loading capacity per unit mass and can complicate formulation scale-up, transport, and storage design [156,157].

### 7.4. Stability Impact of Cyclodextrins

Cyclodextrins (CDs) can either stabilize or destabilize drugs depending on the CD type, drug structure, and complexation conditions. While modified CDs like RMβCD often enhance stability, native CDs, such as βCD, may accelerate degradation, as seen with benzylpenicillin, cefixime, and omeprazole. Factors like pH, degree of methylation, and preparation method also influence the outcome, highlighting the need for case-specific evaluation in formulation design [158].

### 7.5. Toxicological Considerations

The toxicity profiles of cyclodextrins (CDs) vary with the route of administration; for instance, β-CD exhibits nephrotoxicity when administered intravenously due to low water solubility and recrystallization in the kidneys, whereas hydroxypropyl-β-CD (HPβCD) and other derivatives show reduced haemolytic and cytotoxic effects at controlled concentrations, supporting their safer use in mucosal and parenteral formulations [159].

### 7.6. Regulatory Considerations

The regulatory pathway for cyclodextrin-modified lipid nanoparticles remains complex and evolving. Currently, agencies, such as the FDA and EMA, have no specific guidelines for many nanocarrier systems, leading to a case-by-case review with stringent safety and quality requirements [157]. Introducing new excipients or novel uses of excipients like cyclodextrins is treated cautiously, often requiring extensive toxicological and manufacturing data [160]. Although certain CDs, such as HPβCD, are pharmacopeially accepted or GRAS-listed, using them in new nanocarrier systems still demands full Chemistry, Manufacturing, and Controls (CMC) validation. The lack of clear, harmonized nanomedicine guidelines combined with rigorous approval processes can significantly slow CD–NLC development and market translation [157,160].

### 7.7. Cost and Scalability

Pharmaceutical-grade CD derivatives and advanced processing techniques (e.g., high-pressure homogenizers) are costly, potentially limiting widespread use unless therapeutic benefits clearly outweigh production expenses [161].

Looking ahead, the manufacturing methods for CD–SLN/NLCs (e.g., high-pressure homogenization, solvent injection, lyophilization of CD complexes) are adaptations of established processes used for existing products. For example, high-pressure homogenizers are already employed in producing parenteral lipid emulsions and certain nano-formulations. A drug-in-CD-in-NLC formulation would involve an extra step of complexing the drug with a CD (and possibly removing solvent), but is otherwise compatible with scalable techniques. No specific approved drug formulation of this hybrid type exists yet, likely due in part to the need to thoroughly establish safety and reproducibility. However, given the success of lipid nanoparticles and the accepted use of many cyclodextrins (several are pharmacopoeia-listed excipients), a combined CD–lipid nanoparticle product could emerge. Regulatory approval would hinge on demonstrating clear superiority in delivering a challenging drug. One can envision future formulations (for example, an oral nanoparticle for a poorly soluble oncology drug) that utilize a CD inclusion complex within an NLC—effectively merging two excipient strategies to enable a therapy that otherwise has no viable formulation.

## 8. Future Outlook

Cyclodextrin (CD)-modified SLNs and NLCs hold promise for improving the delivery of poorly soluble drugs. Future research should focus on developing smart, stimuli-responsive systems that release drugs in response to pH, enzymes, or redox conditions—particularly valuable for cancer and mucosal therapies. Advances in CD functionalization, such as PEGylation or thiolation, may enhance targeting, mucoadhesion, and circulation time [162]. Sustainable formulation practices using natural lipids, biodegradable surfactants, and solvent-free methods are gaining attention, aligning with regulatory and environmental priorities. Co-delivery strategies and integration with technologies, like hydrogels, microneedles, or 3D printing, expand application potential [163]. Finally, Machine learning and Artificial Intelligence (AI) can aid formulation optimization by predicting optimal component ratios and simulating nanoparticle properties, thereby accelerating development and improving the precision and efficiency of drug delivery systems [164].

## 9. Conclusions

Cyclodextrin-functionalized solid lipid nanoparticles (SLNs) and nanostructured lipid carriers (NLCs) represent a promising and innovative class of drug delivery vehicles. These systems effectively merge the solubilizing and stabilizing functions of cyclodextrins (CDs) with the controlled release behavior, biocompatibility, and site-specific delivery advantages of lipid-based nanocarriers. Evidence from recent studies highlights that incorporating CDs into SLN and NLC structures enhances drug solubility, boosts entrapment efficiency, lowers lipid crystallinity, prolongs drug release, and improves overall stability. These enhancements make CD-lipid conjugates especially suitable for delivering poorly soluble drugs, heat-sensitive molecules, and drugs requiring targeted delivery.

Formulation techniques can be adjusted based on drug characteristics and administration routes, employing strategies such as drug-CD precomplexation, direct embedding, or surface functionalization. Nonetheless, several obstacles remain, including intricate formulation processes, scaling difficulties, potential toxicity from some CD types, and stringent regulatory requirements. These hybrid carriers have been explored for a variety of uses, including oral, dermal, mucosal, oncology, and CNS therapies. To achieve clinical and commercial success, more research is needed to bridge lab findings with real-world applications. Future trends point toward integrating responsive delivery systems, green technologies, personalized therapy approaches, and AI-based design tools. Overall, CD-lipid nanoparticles are a key development in nanomedicine, with continued multidisciplinary efforts essential for their advancement.

## Figures and Tables

**Table 1:** Approved modified cyclodextrins for human pharmaceutical use: chemical modifications, solubility, routes of administration, and applications.

Cyclodextrin Type	Chemical Modification	Approx. Solubility in Water (mg/mL)	Approved Routes of Administration	Example Pharmaceutical Applications	References
HP-β-CD	2-Hydroxypropyl substitution	>600	Oral, IV, Intrathecal	Itraconazole oral/Intravenous (IV) solution (Sporanox^®^), Niemann–Pick Disease Type C, FSGS, vaccines	[91,92]
SBE-β-CD (Captisol^®^)	Sulfobutylether substitution of β-CD	~500	Oral, IV	Voriconazole IV formulation (VFend^®^), Solubilizing agent in marketed injectables (e.g., Voriconazole IV)	[91,93,94]
HP-γ-CD	2-Hydroxypropyl substitution	>500	Ophthalmic	Ophthalmic dexamethasone solutions	[95]
Randomly Methylated β-Cyclodextrin (RAMEB)	Random methylation (mixed methyl ethers)	≥500	Topical (nasal, ocular)	Nasal sprays, eye drops (as excipient)	[96,97,98]
2,6-Di-O-methyl-β-Cyclodextrin (DIMEB)	2,6-Di-O-methyl substitution	~570 mg/mL	Investigational (pharmaceutical excipient)	Production of acellular pertussis vaccine (improving toxin secretion)	[91,99,100]
Sugammadex	γ-CD modified with carboxyl-thio ether side chains	~100 mg/mL (in formulation)	IV	Reversal of neuromuscular blockade (Bridion^®^)	[91]

**Table 2:** Representative therapeutic applications of cyclodextrin-enhanced solid lipid nanoparticles (SLNs) and nanostructured lipid carriers (NLCs).

Application Area	CD Type	Drug/Compound	Carrier Type	Formulation and Benefits	References
Oral (Systemic)	HPβCD	Hydrochlorothiazide	NLC	Enhanced solubility, bioavailability, and sustained diuretic activity in pediatric use	[115]
Ocular (Topical)	HPβCD	Lutein	NLC	Improved corneal distribution and retention, enhanced transcorneal penetration, and formulation stability	[153]
Topical (Dermal)	βCD	Thyme oil (Essential oil)	NLC	Sustained release, enhanced skin permeation, and follicular targeting for topical antimicrobial therapy	[14]
Anticancer (Oral)	Epichlorohydrin-βCD (Poly-βCD)	Altretamine	SLN	Enhanced solubility, high entrapment efficiency, sustained release, and 2.75-fold increase in bioavailability	[103]
Anticancer (Oral)	HPβCD	Paclitaxel	SLN	Enhanced solubility, improved cellular uptake, increased lymphatic delivery, and greater oral bioavailability compared to unmodified solution	[107]

## Data Availability

Data sharing is not applicable to this article as no datasets were generated or analyzed in the current study.

## Abbreviations

API	Active Pharmaceutical Ingredient
BCS	Biopharmaceutics Classification System
CD	Cyclodextrin
SLN	Solid Lipid Nanoparticle
NLC	Nanostructured Lipid Carrier
FT-IR	Fourier Transform Infrared
NMR	Nuclear Magnetic Resonance
ZP	Zeta Potential
HPH	High-Pressure Homogenization
PVA	Polyvinyl Alcohol
PIT	Phase Inversion Temperature
HLB	Hydrophilic–Lipophilic Balance
MAS	Microwave-Assisted Synthesis
UAS	Ultrasound-Assisted Synthesis
SBE-β-CD	Sulfobutylether-β-Cyclodextrin
HP-β-CD	Hydroxypropyl-β-Cyclodextrin
HP-γ-CD	Hydroxypropyl-γ-Cyclodextrin
RAMEB	Randomly Methylated β-Cyclodextrin
DIMEB	2,6-Di-O-methyl-β-Cyclodextrin
IV	Intravenous
DSC	Differential Scanning Calorimetry
TGA	Thermogravimetric Analysis
XRD	X-ray Diffraction
EE	Encapsulation Efficiency
EEn	Emulsification Energy
PSD	Particle Size Distribution
MW	Molecular Weight
PDI	Polydispersity Index
DLS	Dynamic Light Scattering
DL	Drug Loading
UV–VIS	Ultraviolet–Visible
HPLC	High-Performance Liquid Chromatography
USP	United States Pharmacopeia
UWL	Unstirred Water Layer
FDA	Food and Drug Administration
EMA	European Medicines Agency
CMC	Chemistry, Manufacturing, and Controls
GRAS	Generally Recognized as Safe
AI	Artificial Intelligence
SEM	Scanning Electron Microscopy
TEM	Transmission Electron Microscopy
